# Treatment of acute Achilles tendon rupture – a multicentre, non-inferiority analysis

**DOI:** 10.1186/s12891-020-03320-3

**Published:** 2020-06-08

**Authors:** Olof Westin, Tony Sjögren, Simon Svedman, Alexandra Horvath, Eric Hamrin Senorski, Kristian Samuelsson, Paul Ackermann

**Affiliations:** 1 Department of Orthopaedics, Institute of Clinical Sciences, Sahlgrenska Academy, University of Gothenburg, Gothenburg, Sweden; 2 Department of Orthopaedics, Sahlgrenska University Hospital, Mölndal, Sweden; 3 Integrative Orthopedic Laboratory, Department of Molecular Medicine and Surgery, Karolinska Institutet, Stockholm, Sweden; 4 Department of Internal Medicine and Clinical Nutrition, Institute of Medicine, Sahlgrenska Academy, University of Gothenburg, Gothenburg, Sweden; 5 Department of Health and Rehabilitation, Institute of Neuroscience and Physiology, Sahlgrenska Academy, University of Gothenburg, Gothenburg, Sweden; 6 Department of Orthopedic Surgery, Karolinska University Hospital, Stockholm, Sweden

**Keywords:** ATRS, Achilles tendon, Rupture, Treatment, Non-surgical, Surgical treatment, Heel-rise test, Rehabilitation

## Abstract

**Background:**

While numerous clinical studies have compared the surgical and non-surgical treatment of acute Achilles tendon rupture (ATR), there are no studies that have performed a non-inferiority analysis between treatments.

**Methods:**

Data from patients who were included in five randomised controlled trials from two different centres in Sweden were used. Outcomes at 1 year after ATR consisted of the patient-reported Achilles tendon Total Rupture Score (ATRS) and the functional heel-rise tests reported as the limb symmetry index (LSI). The non-inferiority statistical 10% margin was calculated as a reflection of a clinically acceptable disadvantage in ATRS and heel-rise outcome when comparing treatments.

**Results:**

A total of 422 patients (350 males and 72 females) aged between 18 and 71 years, with a mean age of 40.6 (standard deviation 8.6), were included. A total of 363 (86%) patients were treated surgically. The ATRS (difference (Δ) = − 0.253 [95% confidence interval (CI); − 5.673;5.785] *p* = 0.36) and LSI of heel-rise height (difference = 1.43 [95% CI; − 2.43;5.59] *p* = 0.81), total work (difference = 0.686 [95% CI; − 4.520;6.253] *p* = 0.67), concentric power (difference = 2.93 [95% CI; − 6.38;11.90] *p* = 0.063) and repetitions (difference = − 1.30 [95% CI; − 6.32;4.13] *p* = 0.24) resulted in non-inferiority within a Δ − 10% margin for patients treated non-surgically.

**Conclusion:**

The non-surgical treatment of Achilles tendon ruptures is not inferior compared with that of surgery in terms of 1-year patient-reported and functional outcomes.

## Background

Rupture to the Achilles tendon is the most prevalent tendon rupture in the lower extremities, with an increasing incidence, which is estimated at approximately 18 injuries annually per 100,000 individuals [1]. Acute Achilles tendon ruptures are particularly common in male recreational athletes and the injury is associated with persistent deficits in foot and ankle function several years after the initial injury [1]. The general treatment strategy for acute Achilles tendon rupture is either surgical or non-surgical treatment, followed by cast immobilisation or functional bracing and rehabilitation [2]. In spite of this, there is to date no “gold-standard” treatment for Achilles tendon ruptures. While numerous clinical studies compare the two aforementioned treatments, the results are inconsistent with regard to the incidence of complications such as deep venous thrombosis, return to sports and patient-reported outcomes [3]. Several complications, such as infections, adhesion formation, nerve damage and other wound-related complications, are directly related to the surgical treatment of Achilles tendon ruptures [2]. One decisive factor in the selection of treatment is the risk of re-rupturing the Achilles tendon, where surgical treatment has shown a pooled re-rupture rate of 3.5% versus 12.6% in patients treated non-surgically [2, 4].

It has been indicated that patients undergoing surgical treatment for Achilles tendon rupture have superior functional performance in heel-rise tests compared with patients treated non-surgically, possibly owing to a reduced risk of tendon elongation with this treatment [5]. However, there is no study that has used a non-inferiority design to evaluate outcome after treatment in patients who have suffered an acute Achilles tendon rupture.

The advantage of a non-inferior analysis is that it facilitates an understanding of outcomes between two treatments when both are related to benefits and disadvantages. Non-inferior analyses are appropriate if it is possible to demonstrate that one treatment is able to favour outcomes and patients and treating medical professions are willing to sacrifice some degree of benefit in relation to another approved treatment [6]. In terms of this study, a non-inferiority analysis aimed to test whether the outcome of non-surgical treatment was not unacceptably inferior to the outcome of the surgical treatment. This is important, as non-surgical treatment still offers a safer, more cost-effective treatment option, despite having slightly poorer efficacy with regard to a higher incidence of re-rupture and a prolonged time to regain preinjury function compared with the surgical treatment [7]. The non-surgical treatment may therefore still be acceptable due to other advantages if one is willing to sacrifice some degree of benefit relative to the surgical treatment [6].

The purpose of this study was to use non-inferiority analyses to determine whether non-surgical treatment is non-inferior in terms of patient-reported and functional outcomes, compared with surgical treatment after acute Achilles tendon rupture. It was hypothesised that patients in the non-surgical group would not display inferiority in terms of the primary outcomes, the Achilles tendon Total Rupture Score (ATRS) and total work in the heel-rise test, 1 year after the Achilles tendon rupture.

## Methods

### Study participants and eligibility criteria

This study comprised 518 patients who either underwent a surgical or non-surgical treatment following an acute Achilles tendon rupture. Data was collected from five previous randomised controlled trials (RCTs) carried out at two different sites in Sweden; Stockholm and Gothenburg [8–12]. The diagnosis of an Achilles tendon rupture was based upon medical history and clinical examination (tendon palpation and Thompson test). Followingly, patients were randomised regardless of the severity of the rupture. The inclusion and exclusion criteria from the original trials are presented in Table 1. We excluded patients younger than 18 years and those without follow-up outcomes. After applying the exclusion criteria, a total of 422 patients (350 males and 72 females) were included in this study.

**Table 1:** Cohort description

Cohorts	Included (n)	Inclusions	Exclusions	Surgical treatment	Non-surgical treatment	Rehabilitation
Nilsson-Helander et al. [8] (*n* = 97)	88	Clinically verified ATR which was treated within 72 h.	Diabetes mellitus, previous ATR, other lower leg injuries, immunosuppressive therapy and neurovascular diseases.	Seventy-nine patients were treated surgically using the modified Kessler suture technique 8 and 1–0 PDS. The paratenon was carefully repaired and the skin closed with interrupted nylon sutures. Post-operatively, the patients were placed in a below-the-knee cast with the foot in the 30° equinus position.	Fourteen patients were treated immediately after randomisation, with a below-the-knee cast with the foot in the equinus position.	All the patients in both groups were treated with a below-the-knee cast with the foot in the equinus position for 2 weeks, followed by an adjustable angle brace by a physiotherapist for the next 6 weeks. Weightbearing as tolerated was allowed after 6 to 8 weeks.
Olsson et al. [9] (*n* = 100)	87	Clinically verified closed mid-tendon substance rupture treated within 96 h.	Previous ATR, other lower leg injuries, neuromuscular diseases, diabetes mellitus, peripheral vascular disease, immunosuppressive treatment and inability to attend follow-up.	Forty-two patients were operated on using the modified Kessler technique. The tendon was repaired end to end using core suturing with two strong semi-absorbable sutures. No cast was used and the ankle was post-operatively immobilised in a pneumatic walker brace including three heel pads that produce a plantarflexion angle of approximately 22°.	Forty-five patients were treated immediately after randomisation, using the same brace as in the surgical group, including the three heel pads.	Patients were allowed full weightbearing, which was encouraged from the first post-operative day for both groups. Early active rehabilitation started 2 weeks post-operatively and included both range of motion and strength training following a standardised protocol. The surgical group was mobilized in the brace for 6 weeks and the non-surgical group for 8 weeks.
Aufwerber et al. [12] (*n* = 150)	103	Clinically verified ATR which was treated within one week.	Ongoing anticoagulation treatment, known kidney failure, heart failure with pitting oedema, thrombophlebitis, thromboembolic event during the previous 3 months, known malignancy, haemophilia, pregnancy, other surgery during the previous month, inability to follow instructions and planned follow-up at another hospital.	One hundred and three patients were operated on surgically using the modified Kessler suture technique with two 1–0 polydioxanone (PDS II) sutures. The paratenon and fascia cruris were then sutured separately using 3–0 Vicryl (Ethicon). After surgery the patients were prospectively randomised into two groups, a full-weight-bearing and non-weight-bearing rehabilitation regime.	Full-weight-bearing group: after surgery, a walker orthosis with an adjustable angle of motion was used for the next 6 weeks. Functional mobilisation with one-hour daily motion exercise was initiated directly post-operatively. Non-weight-bearing group: received a conventional non-weight-bearing below-knee plaster cast with the ankle in a 30° equinus position. At 2 weeks post-operatively, the cast was replaced by a removable walker orthosis with three heel wedges for the remaining 4 weeks of immobilisation. Every consecutive week, a heel wedge was removed. At 6 weeks, all patients discontinued immobilization.	
Domeij-Arverud et al. [11] (*n* = 26)	25	Clinically verified ATR which was treated within 72 h.	Same as Aufwerber et al. [12]	Twenty-five patients were operated surgically using the same techniques as Valkering et al. [13] All patients received a below-knee plaster cast with the ankle in 30° equinus and were non-weight-bearing with crutches during the first 2 weeks. After surgery the patients were prospectively randomised into two groups, both groups non-weight-bearing, but the intervention group received adjuvant foot intermittent pneumatic compression.	All patients received a below-knee plaster cast with the ankle in 30° equinus in the outpatient clinic shortly after the completion of surgery, and were non-weight-bearing with crutches during the first 2 weeks. The intervention group received intermittent pneumatic compression applied to the foot under the plaster cast, which was discontinued at 2 weeks post-operatively. At the 2-week visit all patients received a lower leg orthosis and were instructed to start full weight-bearing. The orthotic treatment was discontinued at 6 weeks post-operatively.	
Domeij-Arverud et al. [10] (*n* = 150)	119	Clinically verified ATR which was treated within 96 h.	Same as Aufwerber et al. [12]	One hundred and nineteen patients were operated surgically using the same techniques as Valkering et al. [13] After surgery the patients were prospectively randomised into two groups both groups non-weight-bearing, but the intervention group received adjuvant calf intermittent pneumatic compression.	Control group: The patients received a below-knee plaster cast applied in the outpatient clinic shortly after the completion of surgery, with the ankle plantar-flexed to provide 30° of equinus. Patients were non-weight-bearing during the first 2 post-operative weeks and were given crutches. Intervention group: The intervention group received intermittent pneumatic compression applied to the calf beneath a walker orthosis. At 2 weeks post-operatively the intervention was discontinued. At the 2-week visit all patients received a lower leg orthosis and were instructed to start full weight-bearing. The orthotic treatment was discontinued at 6 weeks post-operatively.	

*Clinically verified Achilles tendon rupture (ATR): presenting with symptoms including sudden increase in pain around the Achilles, weakness and poor balance and limited walking distance* [14] *and a palpable gap in the tendon and a positive Thompson test* [15]

*PDS –* polydioxanone

### Follow-up and clinical evaluation

All the patients were evaluated by experienced physiotherapists 1 year after the surgical or non-surgical treatment of Achilles tendon rupture. The ATRS was used to reflect the patient’s perception of Achilles function [14, 16]. The objective evaluation consisted of testing foot and ankle function on both limbs by using a test battery of heel-rise tests to determine concentric power and muscular endurance. Muscular endurance tests consisted of total work, maximum repetitions and heel-rise height. The tests were recorded using a linear encoder with MuscleLab (Ergotest Technology, Oslo, Norway) software. This software is able to record data from different type of sensors connected to the MuscleLab. The testing protocol was conducted as previously described in the literature [8, 15, 16]. All the results were reported as the limb symmetry index (LSI). The LSI reflects relative limb strength or function and is calculated by dividing the test score for the affected limb by that of the healthy limb and multiplying by 100 to obtain side-to-side differences expressed as a percentage [16].

### Achilles tendon total rupture score

The ATRS [3] is a patient-reported outcome which is valid, reliable and sensitive for evaluating Achilles function in patients with an Achilles tendon rupture (intraclass correlation coefficient (ICC) = 0.96) [16]. The ATRS consists of 10 questions, each scored from 0 (worst) to 10 (best), with a maximum total score of 100. A higher score indicates less physical disability and a higher quality of life [3]. This study used the original version of the ATRS instrument as described by Nilsson-Helander et al. [3].

### Heel-rise test: concentric power

The heel-rise test for concentric power was performed with the patient standing on one leg in a weight machine doing single-leg heel-rises as quickly and forcefully as possible. During this procedure, the knee is not allowed to flex more than 20° and the procedure is performed in three intervals. The patients started with 13 kg and an additional 10 kg was added at each interval until a decrease in power output was recorded. A linear encoder, connected to MuscleLab software, was attached to the shoe and the best trial (i.e. with the highest force in watts (W)) for each weight was recorded and used for analysis. This was calculated by entering the weight of the patient and the extra external weight into the MuscleLab software for which peak power was calculated for each weight interval. For the patients included from the cohorts of Domeij-Arverud [10, 11] the heel-rise test for concentric power was performed without adding extra weight [17].

### Heel-rise test: total work, maximum repetitions and height

The heel-rise test reflects the function of the plantar flexors of the lower limb and was evaluated by assessing single-leg height, repetitions and total work (in joules (J)). The uninjured limb was always tested first. The test was performed with the patient standing on a 20 cm flat box with a tilted wedge (10°). The patients were instructed to go as high as possible on each heel rise and perform as many heel rises as possible, while a metronome was used to keep the frequency at 30 heel rises a minute. The test was terminated when the patient was unable to maintain the frequency or did not perform a correct heel rise (minimum 2 cm). A linear encoder (MuscleLab) was used to measure the aforementioned outcomes. With regard to the heel-rise height, the maximum height achieved by the patient was recorded for data analysis. To obtain the values for total work, this was calculated as body weight x total distance in J.

### Statistical analysis

All the statistical analyses were performed using the Statistical Analysis System (SAS, SAS institute Inc., NC, USA). Continuous variables were described as the mean (standard deviation (SD)), median (minimum and maximum) and categorical variables with count (n) and proportions (%).

The heel-rise tests and ATRS were compared between the two treatment groups with the Mann-Whitney U-test and reported with *p*-values and confidence intervals (CI). To calculate confidence intervals for the continuous variables, bootstrapping of 1000 replicated picking the 2.5 and 97.5 percentiles of the 10,000 mean differences was used. To determine non-inferiority, we hypothesised that non-surgical treatment would not be inferior to surgical treatment by delta-Δ margin (difference between groups) for the ATRS±10 points and LSI ± 10% which have been clinically accepted as non-inferiority and preclude equality by using a T-test, 95% CI and *p*-value. The minimal clinical important difference (MCID) of ATRS has been stated as 10 points which equals 10% of the total score (100 points) and is in line with previous literature on the topic [3]. At the 1-year follow-up, confounders adjusted for were age, sex, body mass index (BMI) defined as (kg/m^2^) and smoking (yes/no). All testing was performed at a 5% significance level.

## Results

In the present study, 422 patients were included, whereof 363 were treated surgically and 59 non-surgically. The patients were between 18 and 71 years of age, with a mean age of 40.6 (SD 8.6). The mean BMI of the included patients was 26.1 (SD 3.3) and 95.2% were non-smokers. Demographic differences between patients that received surgical and non-surgical treatment are presented in Table 2. A larger proportion of males were treated surgically (85.1%) compared with females (69.5%) (*p* = 0.0031). In addition, there was a larger number of males in the cohort compared with females, 350 (82.9%) versus 72 (17.1%) respectively. This discrepancy between sexes also resulted in significant differences in the mean height (*p* = 0.0044) and weight (*p* = 0.0024) of the patients for the two treatments. Nevertheless, there were no differences in BMI (*p* = 0.060), age (*p* = 0.33) and smoking (*p* = 0.17) between the two treatment options. The prevalence of smoking was rare, with only 16 patients being smokers, and, of these, the majority were treated surgically, 12 versus four in the non-surgical group.

**Table 2:** Patient baseline characteristics

Demographics	Total included cohort (***n*** = 422)	Surgical treatment (***n*** = 363)	Non-surgical treatment (***n*** = 59)	*p*-value
**Patient sex**				
Males	350 (82.9%)	309 (85.1%)	41 (69.5%)	
Females	72 (17.1%)	54 (14.9%)	18 (30.5%)	0.0031
**Age (years)**	40.6 (8.6) 40.0 (18.0; 71.0) *n* = 422	40.4 (8.4) 39.0 (18.0; 71.0) *n* = 363	41.7 (9.6) 41.0 (21.0; 64.0) *n* = 59	0.33
**Height (cm)**	178.3 (8.7) 180.0 (144.0; 200.0) *n* = 412	178.8 (8.4) 180.0 (144.0; 200.0) *n* = 354	175.1 (9.7) 175.5 (153.0; 195.0) *n* = 58	0.0044
**Weight (kg)**	83.1 (13.2) 83.0 (43.0; 129.0) *n* = 413	83.8 (13.1) 84.0 (43.0; 129.0) *n* = 355	78.4 (12.7) 75.0 (57.0; 112.0) *n* = 58	0.0024
BMI (kg/m^2^)	26.1 (3.3) 25.7 (19.6; 43.6) *n* = 412	26.1 (3.3) 25.7 (19.6; 43.6) *n* = 354	25.5 (3.2) 24.6 (20.1; 38.9) *n* = 58	0.060
**Smoking**				
Non-smoker	316 (95.2%)	275 (95.8%)	41 (91.1%)	
Smoker	16 (4.8%)	12 (4.2%)	4 (8.9%)	0.17
**Study**				
Nilson-Helander et al. [8] (*n* = 97)	88 (20.9%)	74 (20.4%)	14 (23.7%)	
Olsson et al. [9] (*n* = 100)	87 (20.6%)	42 (11.6%)	45 (76.3%)	
Aufwerber et al. [12] (*n* = 150)	103 (24.4%)	103 (28.3%)	0	
Domeij-Arverud et al. [11] (*n* = 26)	25 (5.9%)	25 (6.9%)	0	
Domeij-Arverud et al. [10] (*n* = 150)	119 (28.2%)	119 (32.8%)	0	

*For categorical variables, n (%) is presented. For continuous variables, the mean (standard deviation (SD))/median (min; max)/n = is presented*

*BMI* Body mass index

### Achilles tendon rupture score

**Fig. 1:**
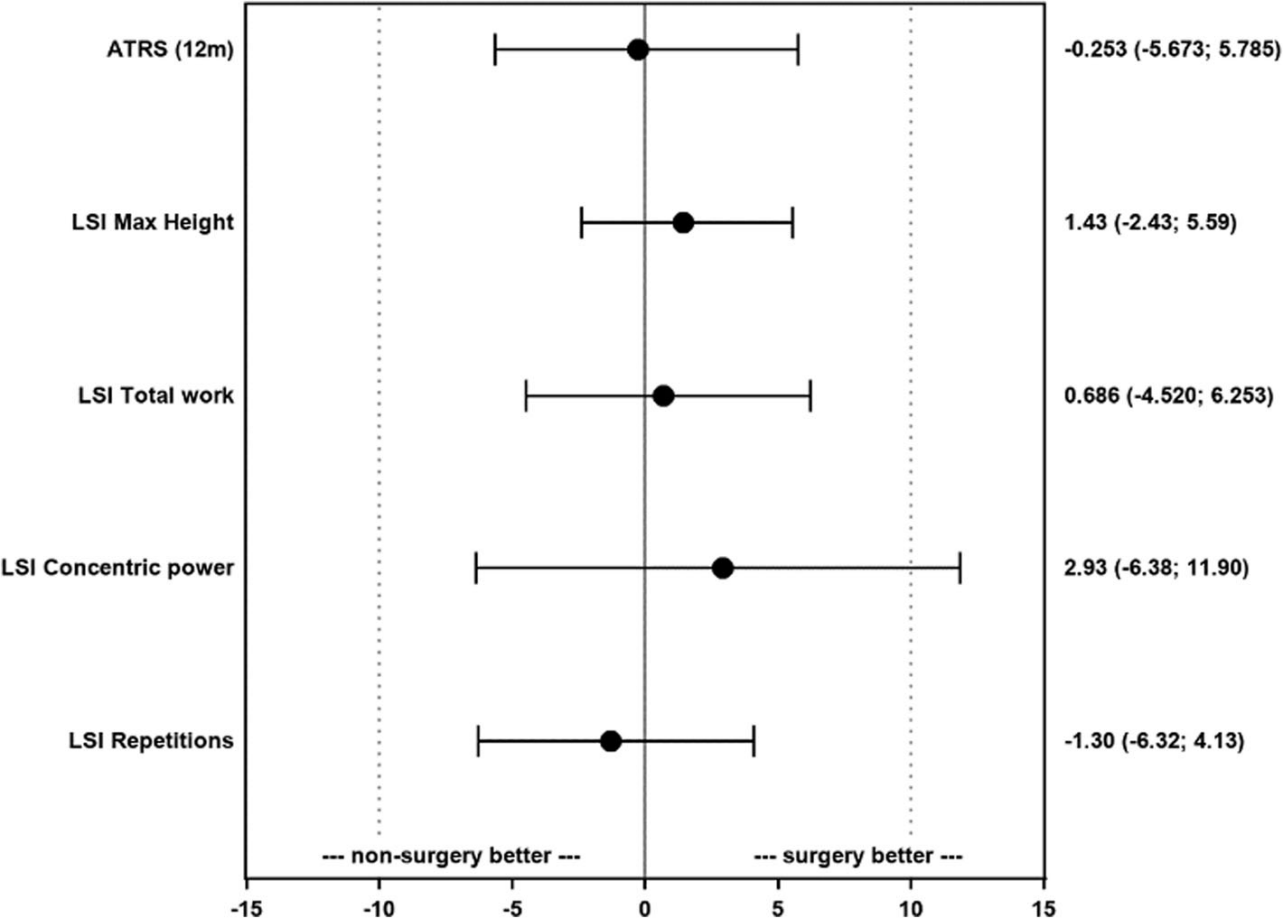
Non-inferior analysis comparing non-surgical treatment with surgical treatment. Non-inferior margin: *±10% Δ. ATRS: Achilles tendon rupture score. LSI: Limb symmetry index. Numbers present: difference, 95% confidence intervals*

In terms of the ATRS, the mean for both groups at 1 year was 81.5 (SD 18.6) points. The surgically treated patients scored 81.5 (SD 18.2) compared with 81.7 (SD 21.4) for the non-surgical group (difference = − 0.25 [95% CI; − 5.67; 5.79] *p* = 0.36) (Table 3;Fig. 1).

**Table 3:** Results of 12-month postinjury evaluation

Outcome	Total (***n*** = 422)	Surgical treatment (***n*** = 363)	Non-surgical treatment (***n*** = 59)	*p*-value	Difference between groups Mean (95% CI)
**Achilles Tendon Rupture Score**	81.5 (18.6) 87.0 (0.0; 100.0) *n* = 399	81.5 (18.2) 87.0 (0.0; 100.0) *n* = 340	81.7 (21.4) 91.0 (2.0; 100.0) *n* = 59	0.36	−0.253 (−5.673; 5.785)
**LSI – maximum height**	80.7 (15.3) 81.1 (28.0; 139.3) *n* = 397	80.9 (15.5) 81.3 (33.6; 139.3) *n* = 338	79.5 (14.4) 79.6 (28.0; 105.0) *n* = 59	0.81	1.43 (−2.43; 5.59)
**LSI – total work**	73.3 (25.3) 73.2 (7.0; 288.0) *n* = 396	73.4 (26.4) 73.1 (7.0; 288.0) *n* = 337	72.7 (18.2) 75.0 (7.0; 111.4) *n* = 59	0.67	0.686 (−4.520; 6.253)
**LSI – concentric power**	85.0 (27.1) 80.6 (24.6; 216.0) *n* = 390	85.4 (25.7) 82.0 (24.6; 200.0) *n* = 332	82.5 (34.1) 72.9 (34.0; 216.0) *n* = 58	0.063	2.93 (−6.38; 11.90)
**LSI – repetitions**	90.3 (23.6) 90.2 (13.3; 275.0) *n* = 397	90.1 (24.5) 89.2 (13.3; 275.0) *n* = 338	91.4 (17.9) 92.0 (26.0; 139.4) *n* = 59	0.24	−1.30 (−6.32; 4.13)

*For continuous variables, the mean (standard deviation)/median (min; max)/n = is presented*

*CI* Confidence Interval*, LSI* Limb Symmetry Index

### Maximum heel-rise height

The 1-year limb symmetry index (LSI) for heel-rise height was a mean of 80.7% (SD 15.3). The LSI for heel-rise height did not differ between groups. The surgically treated group had a mean LSI for heel-rise height of 80.9%, while the non-surgical group had 79.5% (difference = 1.43 [95% CI; − 2.43; 5.59] *p* = 0.81) (Table 3;Fig. 1).

### Heel-rise total work

The mean LSI for heel-rise total work at the 1-year follow-up was 73.3% (SD 25.3). There was no difference between the surgical and non-surgical treatment groups in the 1-year LSI for heel-rise total work. The mean for the surgically treated patients was 73.4%, while it was 72.7% for the non-surgical group (difference = 0.686 [95% CI; − 4.520; 6.253] *p* = 0.67) (Table 3;Fig. 1).

### Heel-rise concentric power

The mean 1-year LSI for heel-rise concentric power was 85.0% (SD 27.1). There was no difference in the mean values for the LSI of heel-rise concentric power in patients treated surgically or non-surgically. The mean LSI of heel-rise concentric power was 85.4% for surgically treated patients and 82.5% for the non-surgical group (difference = 2.93 [95% CI; − 6.38; 11.90] *p* = 0.063) (Table 3;Fig. 1).

### Heel-rise repetitions

The mean 1-year LSI for heel-rise repetitions was 90.3% (SD 23.6). For the surgically treated patients, the mean was 90.1%, while it was 91.4% for the non-surgical group, which did not differ between the treatment groups (difference = − 1.30 [95% CI; − 6.32; 4.13] *p* = 0.24) (Table 3;Fig. 1).

## Discussion

This randomised, non-inferior study based on a pooled sample of 422 patients is the first investigation to demonstrate that non-surgical treatment is non-inferior to surgical treatment 1 year after Achilles tendon rupture with regard to the ATRS and LSI in the heel-rise tests for concentric power, heel-rise height, total work and heel-rise repetitions.

The non-inferior evaluation made it possible to encourage risk-benefit assessments and safety advantages that resulted in a marginal, clinically acceptable loss of efficacy in heel rise and ATRS. The results of this study may help when deciding on the treatment method after Achilles tendon rupture and, ultimately, adverse complications related to surgical treatment could potentially be avoided [4, 18]. For this reason, the present study confirms that non-surgical treatment is an acceptable alternative for the treatment of acute Achilles tendon rupture in terms of functional outcome and ATRS. This is in line with previous findings indicating that surgical treatment does not necessarily produce a superior outcome in terms of strength and range of motion in comparison with non-surgical treatment [19].

The findings in this study suggest non-inferiority using the fixed-margin method, calculating the lower limit of 95% CI difference between treatments. Conclusions can be drawn if the CI lies within the margin of delta-Δ. To preserve viability, our fixed (delta-Δ) margin was set at 10%, a narrow margin determined by a Delphi-type approach [20]. The delta-Δ is determined by statistical and clinical judgement, i.e. asking orthopaedic surgeons and physiotherapists how much benefit over side-effects they are willing to forego by using non-surgical treatment. In order not to depend completely on the empiricism of investigators and the expectations of the medical community, the ATRS was used in the non-inferior analysis to incorporate patients’ perceptions of treatment outcome. The ATRS is an important outcome tool, which enables the creation of an acceptable margin based on anticipated benefits and risks. The results suggest non-inferiority that approaches equivalence for the ATRS at the 1-year follow-up, similar to that found in other studies, indicating that no difference exists with regard to the ATRS between patients undergoing surgical and non-surgical treatment [8, 9, 21]. This outcome suggests that restrictions caused by symptoms in physical activity and everyday living are not inferior in non-surgically treated Achilles tendon rupture patients as opposed to those treated surgically.

Our results further demonstrate that maximum heel-rise height is non-inferior for patients treated non-surgically, which is in line with the results of previous studies [22–25]. A reduction in the maximum heel-rise height performance has been associated with tendon elongation, creating abnormalities in power generation around the ankle and in gait [5, 26]. A meta-analysis by Jiang et al. [27] comprising 894 patients revealed no difference in tendon elongation outcome between non-surgical and surgical treatment after Achilles tendon rupture. Nevertheless, it is important to acknowledge that recent studies have associated tendon elongation preceding poor maximum heel-rise performance with potentially faulty rehabilitation [28]. Erroneous rehabilitation may be produced by overstraining the tendon and the degree of tendon end separation in non-surgical treatment. For this reason, the non-surgical treatment of Achilles tendon ruptures requires an effective rehabilitation plan and implementation. The cause and effect of tendon elongation have, however, not been established. In addition, outcomes of tendon elongation measurements are not consistent between different methods [29, 30].

Traditional rehabilitation protocols involved the usage of a below-knee non-weight bearing rigid cast for 6 weeks, followed by mobilization of the ankle joint and strengthening exercises [31]. However, this rehabilitation regimen has been questioned as recent investigations have demonstrated that functional rehabilitation including postoperative early weightbearing combined with early ankle motion exercises are associated with earlier return to sport, increased patient satisfaction and reduced tendon elongation when compared with traditional rehabilitation protocols [32–34]. This demonstrate that the type of rehabilitation and onset of load is imperative and can moderate effectiveness of treatment following the initial treatment of ATR. In general, all patients in our cohort that were surgically treated discontinued with a walker orthosis after 6 weeks post-operative, while the non-surgically treated patients discontinued their walker orthosis at 8 weeks post-operative. In some of the trials, the rehabilitation within the first 2 weeks differed, although this had little effect on the postoperative outcome including the ATRS and function heel-rise tests.

The 1-year results for concentric power recorded during heel-rise evaluation resulted in a confidence interval of (CI; -6.38;11.90). The lower margin of the confidence interval is above the lower Δ-margin but exceeds the upper Δ-margin (10%) non-significantly in favour of surgical treatment. This is in agreement with a meta-analysis by Ke Zouh et al. [35] which reported comparable numbers when comparing surgical with non-surgical treatment with regard to concentric power (CI; -2.59;17.06, *p* = 0.15) [35]. This finding is, however, contradicted by one of our included cohorts [8]. Nilsson-Helander et al. [8] presented significantly superior results for heel-rise concentric power at the 6-month follow-up for patients treated surgically. Nevertheless, there is still a significant decrease in function relative to the uninjured leg in the included cohorts [8].

Herein, we demonstrate that the heel-rise test for total work and repetitions resulted in non-inferiority for the non-surgical treatment. A randomised controlled trial comprising 80 patients allocated to non-surgical and surgical treatment found no significant differences in total work and repetitions at the 12-month follow-up [36]. In contrast, Lantto et al. [37] reported that plantar flexion strength is about 10% less for patients treated non-surgically, while another trial found significantly higher plantar flexion strength in patients undergoing non-surgical treatment [38]. It is important to be aware that heel-rise work and repetition reflect muscular strength and endurance and may vary due to individual variations in muscle mass, body habitus, patients’ physical condition prior to injury and the way patients pursue their former physical activity and not the initial treatment itself. A reduction in plantar flexion strength may not be clinically relevant for most patients, but it is still a common argument that is used to endorse the fact that surgery should be considered for athletic patients with an Achilles tendon rupture. This argument has, however, been challenged by studies of National Basketball Association and National Football League players in which the majority of players did not regain their pre-injury level performance or were unable to return to sport following surgical Achilles tendon repair [38, 39].

Furthermore, controversy exists regarding the best treatment strategy for acute Achilles tendon rupture and whether adverse events such as deep venous thrombosis are comparable between treatments. Decreased re-rupture rates after surgical treatment have been the main argument in promoting surgery as the primary treatment option compared with non-surgical treatment [40], as the former treatment approach results in a re-rupture rate of 3.5% and the latter in a rate of 12.6% with a pooled relative risk of 0.27 (95% CI; 0.11;0.64) [4]. However, a meta-analysis pertaining to the outcomes following surgical and non-surgical treatment of ATR indicated that, when early range of motion was incorporated in the treatment regimen, there was no difference in the re-rupture risk between the two treatments [41]. As a result, an effective rehabilitation protocol for Achilles tendon rupture appears to be more important than the surgical treatment method itself. In addition, surgical repair involves, but is not limited to, an increased risk of infection, adhesions and disturbed skin sensibility with a pooled relative risk of 10.6 (95% CI; 4.8;23.3) as compared with non-surgical treatment [4]. Damage to the sural nerve during surgical repair of an ATR has also been associated with increased risk of postoperative pain and reduced function [18]. Moreover, a recent investigation of direct health-care costs and indirect costs (sick leave days) showed that surgical treatment was more expensive compared with non-surgical treatment [7].

The complications and benefits reported after both surgical and non-surgical treatments indicate that there is an intricate relationship between patient-related factors and treatment approach. Our results extend previous knowledge, suggesting non-inferiority for non-surgical treatment in evaluations of heel-rise performance and ATRS. Non-surgical treatment with an appropriate rehabilitation strategy may result in acceptable functional outcome, re-rupture risk and lower general costs, without the risk of wound complications. Including the patients’ preferences and expectations in relation to clinical outcome is therefore imperative when deciding on treatment approach. Patient compliance is, however, critical to the success of non-surgical interventions and larger high-quality multicentre studies are required to determine the optimal treatment [42, 43].

### Limitations of this study

Considering the nature of and the differences between non-surgical and surgical treatment options for acute Achilles tendon rupture, the blinding of investigators, participants and outcome assessors was not practicable when personnel needed to observe participants in the follow-up process. Potentially influencing imprecise end-point ascertainment, as well as non-blinded patients, may have influenced their behaviour and response to outcome, thereby generating performance bias [44]. However, primary outcome was also assessed by a patient-questionnaire (ATRS) as primary evidence of valuable life-functional restrictions and pain. This could increase the magnitude of detection bias, because patient estimations are a subjective measurement [45]. Nevertheless, the ATRS outcome tool in our study is similar to that in other studies comparing alternative treatments [8, 9, 21].

The number of patients included in the non-surgical treatment group was limited to 59 compared with 363 who were treated surgically. The unevenly distributed number of patients might have had an impact on the analysis, with a substantial effect size in the evaluation of functional outcome that may bias the overall results as compared with treatment. However, the two treatment groups were comparable with regard to demographic characteristics including age and BMI. Despite the uneven number of patients, surprisingly good results were obtained in relation to heel-rise repetitions after non-surgical treatment compared with surgical treatment. Finally, the cohorts used in this study had different treatment interventions regarding surgical technique, exclusion criteria and rehabilitation regimen. This may have created an imbalance in treatment adherence and may have impacted the overall consistency, especially as the non-surgical treatment group consisted of a small study sample.

## Conclusion

The non-surgical treatment of Achilles tendon ruptures is non-inferior compared with surgery at 1 year in terms of the ATRS and LSI for heel-rise height, total work, repetitions and concentric power.

## Data Availability

The datasets used and/or analysed during the current study are available from the corresponding author on reasonable request.

## Abbreviations

ATR: Achilles Tendon Rupture
ATRS: Achilles Tendon Total Rupture Score
BMI: Body Mass Index
CI: Confidence Interval
ICC: Intraclass Correlation Coefficient
J: Joule
LSI: Limb Symmetry Index
RCT: Randomised Controlled Trial
SD: Standard Deviation
W: Watt
