# Correction to: Treatment of acute Achilles tendon rupture - a multicentre, non-inferiority analysis

**DOI:** 10.1186/s12891-021-04045-7

**Published:** 2021-02-16

**Authors:** Olof Westin, Tony Sjögren, Simon Svedman, Alexandra Horvath, Eric Hamrin Senorski, Kristian Samuelsson, Paul Ackermann

**Affiliations:** 1 Department of Orthopaedics, Institute of Clinical Sciences, Sahlgrenska Academy, University of Gothenburg, Gothenburg, Sweden; 2 Department of Orthopaedics, Sahlgrenska University Hospital, Mölndal, Sweden; 3 Department of Molecular Medicine and Surgery, Karolinska Institutet, Integrative Orthopedic Laboratory, Stockholm, Sweden; 4 Department of Internal Medicine and Clinical Nutrition, Institute of Medicine, Sahlgrenska Academy, University of Gothenburg, Gothenburg, Sweden; 5 Department of Health and Rehabilitation, Institute of Neuroscience and Physiology, Sahlgrenska Academy, University of Gothenburg, Gothenburg, Sweden; 6 Department of Orthopedic Surgery, Karolinska University Hospital, Stockholm, Sweden

**Correction to: BMC Musculoskelet Disord 21, 358 (2020)**

**https://doi.org/10.1186/s12891-020-03320-3**

Following publication of this article [1], the authors report the following Corrections to the main text:

i) The authors noticed that the reported number of patients that were treated surgically were too many (*n* = 363) and have corrected the numbers (*n* = 337). Patients from the cohort Nilsson-Helander et al. [2] were incorrectly labeled as surgically treated when they had received non-surgical treatment. In view of this database mix up, the statistical analysis was re-performed with the correct database and the results changed accordingly.
ii) With updated guidelines on how to perform non-inferiority studies having been published in the *New England Journal of Medicine* [3], since the original analysis was published, the statistical method have accordingly been changed to using Fisher’s Non-parametric Permutation Test. The limb symmetry index for total work is inconclusive. The main conclusion, however, is unchanged that there is no significant difference between the surgery and non-surgery.
